# Early Continence and Erectile Function Recovery Following Transvesical Single-Port Robot-Assisted Radical Prostatectomy: Initial Single Institution Experience

**DOI:** 10.3390/cancers17172793

**Published:** 2025-08-27

**Authors:** Brandon L. Ward, Anthony Y. Zhang, Michael S. Leapman, Jaime A. Cavallo, Isaac Y. Kim

**Affiliations:** 1 Yale School of Medicine, Yale University, New Haven, CT 06492, USA; brandon.ward@yale.edu (B.L.W.); anthony.zhang@yale.edu (A.Y.Z.); michael.leapman@yale.edu (M.S.L.); jaime.cavallo@yale.edu (J.A.C.); 2 School of Medicine, Case Western Reserve University, Cleveland, OH 44106, USA; 3 School of Medicine, University of Connecticut, Farmington, CT 06030, USA; 4 Veterans Affairs Connecticut Healthcare System, West Haven, CT 06516, USA

**Keywords:** robot-assisted prostatectomy, single-port robotic surgery, minimally invasive surgical procedures, prostate cancer, urinary incontinence, erectile dysfunction

## Abstract

**Background/Objectives**: Early recovery of urinary continence and sexual function remains a key focus following radical prostatectomy. The transvesical single-port robot-assisted radical prostatectomy (SP-TV-RARP) approach is a novel technique with very limited evidence that has been suggested to preserve the Retzius space and allow extraperitoneal access through the bladder. We aimed to evaluate early functional and oncologic outcomes following SP-TV-RARP at a single academic institution. **Methods**: We retrospectively reviewed 21 patients who underwent SP-TV-RARP by a single surgeon between September 2024 and May 2025. Continence is defined as being pad-free, and return of erectile function is defined as having erections sufficient for penetration. Functional and oncologic outcomes were assessed using clinical follow-up documentation and analyzed with Kaplan–Meier analysis. **Results**: The median patient age was 65 years, and 52.4% had pT3 disease. Positive surgical margins were observed in 62% overall and 30% in men with organ-confined disease. No intraoperative or 30-day postoperative complications occurred. Approximately 43% of patients achieved continence within one day of urethral catheter removal, with 75% of patients being pad-free at 3 months. Median SHIM and AUA-SS scores did not significantly decline at 3 months compared to baseline. The median time to recovery of erectile function was 69 days, and 67% recovered at the last follow-up. Biochemical recurrence occurred in 2 patients (15.4%) within 6 months. **Conclusions**: SP-TV-RARP appears safe and may facilitate early return of urinary continence and erectile function. Although inferior oncologic outcome is a potential concern during early adoption, functional outcomes were favorable. Further prospective evaluation is warranted to confirm long-term oncologic efficacy.

## Simple Summary

Surgical removal of the prostate (radical prostatectomy) is the most frequently chosen treatment option for prostate cancer. However, this procedure can lead to side effects such as urine leakage and difficulty with erections. A newer surgical technique, called single-port transvesical robot-assisted radical prostatectomy, uses a single incision through the bladder and aims to minimize disruption to the tissues surrounding the prostate. In this study, we evaluated short-term outcomes in men who underwent this procedure at our institution. Most patients regained bladder control and sexual function within three months, with no major complications. These early findings suggest that this approach may support faster recovery, even during the initial adoption phase. Further research is needed to assess long-term cancer control and the broader applicability of this technique in clinical practice.

## 1. Introduction

Radical prostatectomy (RP) remains a standard treatment for localized prostate cancer and is increasingly performed by the robot-assisted approach, now representing over 80% of cases in the United States [1,2]. While traditional multi-port robot-assisted radical prostatectomy (MP-RARP) achieves excellent long-term oncologic and functional outcomes, attention has shifted towards strategies that accelerate the recovery of urinary continence and erectile function, two major determinants of postoperative quality of life after prostatectomy [3,4,5].

Single-port robotic-assisted radical prostatectomy (SP-RARP) has emerged as a technique aimed at minimizing anatomical disruption, reducing incisions required, and potentially improving perioperative recovery [6,7,8]. Comparative studies and meta-analyses report reduced postoperative pain, shorter hospital stays, and earlier catheter removal with SP-RARP compared to MP-RARP, without compromising continence, potency, or oncologic control [9,10,11,12,13]. Among the SP-RARP techniques, the Retzius-sparing approach has been most widely studied, demonstrating earlier continence recovery in randomized and observational studies [4,5,14].

The transvesical SP-RARP (SP-TV-RARP) adapts Retzius-sparing principles by entering through the bladder, maintaining its natural position, preserving the Retzius space, and avoiding peritoneal entry [6,14,15,16]. A key technical element is the use of laparoscopy with pneumovesicum (LPV), in which the bladder is insufflated with gas to create the working space, thus avoiding violation of the peritoneal cavity, reducing intra-abdominal pressure, and potentially preserving pelvic and renal hemodynamics [14,16]. Early single-institution series have demonstrated encouraging functional outcomes, with 75–91% of patients achieving urinary continence (use of 0–1 pads per day) within days of urethral catheter removal [6,15,17] and erectile function recovery rates exceeding those typically reported for multiport techniques [6,15]. Preliminary oncologic results from SP-TV-RARP suggest positive surgical margin and biochemical recurrence rates comparable to early single-port series, while data from broader SP-RARP experiences indicate similar oncologic control to multiport approaches [12,13,18].

Although previous studies have shown promising data on the benefits of the extraperitoneal transvesical single-port approach in efficacy and improved outcomes for localized prostate cancer, there remains limited data elucidating the full extent of functional recovery benefits of this technique, with most current literature coming from a single institution. Therefore, the aim of this study is to evaluate early functional and oncologic outcomes of transvesical single-port RARP at a separate academic institution. We hypothesize that the SP-TV-RARP will result in earlier recovery of urinary continence and erectile function as compared to multi-port robotic approaches in the literature. By reporting outcomes from an institution outside the pioneering centers and including a broader patient risk spectrum, this study adds to the limited evidence on SP-TV-RARP and provides additional data on adopting the technique.

## 2. Materials and Methods

We retrospectively analyzed all patients who underwent single-port transvesical robot-assisted radical prostatectomy (SP-TV-RARP) by a single surgeon at our institution between September 2024 and May 2025, using a prostatectomy outcomes database that is updated prospectively. Prospectively recorded variables included patient age, body mass index (BMI), preoperative prostate-specific antigen (PSA) level, serum hemoglobin, serum creatinine, serum testosterone, prostate volume measured from magnetic resonance imaging (MRI), PIRADS score, Gleason score, Grade Group, and baseline American Urological Association Symptom Score (AUA-SS) and Sexual Health Inventory for Men (SHIM) score collected at the preoperative visit. Additional prospectively recorded variables included date of surgery, surgical approach, operative time, estimated blood loss (EBL), pathology findings (prostate weight, pathologic stage, surgical margin status, extraprostatic extension, seminal vesicle invasion, perineural invasion, and lymphovascular invasion), and thirty-day postoperative complications as identified by our institutional automated abstraction algorithm [19]. These complications included operating room return, readmission, mortality, urinary tract infection, urine leak, bladder neck contracture, pelvic fluid collection, lymphocele, sepsis, cardiac events, pneumonia, unplanned intubation, prolonged ventilator use, bowel injury, prolonged nasogastric tube (NGT) or nil per os (NPO) status, *Clostridioides difficile* infection, and stroke. All patients had their urethral catheter removed seven days after surgery and were scheduled for follow-up visits at approximately three, four, and six months postoperatively, although some patients were seen earlier if clinically indicated.

For this study, we supplemented these data through retrospective chart review to collect variables not routinely included in the prospective database. These included postoperative PSA levels, date of biochemical recurrence (BCR), receipt of salvage therapy, date of return to urinary continence, number of pads per day, date of return of erections firm enough for penetration, use of phosphodiesterase type 5 inhibitors (PDE5i), and any missing baseline demographic, laboratory, or imaging values.

Patients were ineligible for this approach if prostate volume was greater than 60 mL on preoperative MRI or if they opted for lymph node dissection after discussion with the surgeon. Patients with incomplete follow-up or missing key variables were excluded from analyses in which those data were essential. In addition to SHIM and AUA-SS scores, key variables included baseline demographic, laboratory, and imaging parameters (e.g., PSA, prostate volume, and PIRADS score) as well as postoperative oncologic outcomes such as PSA follow-up, pathologic stage, and surgical margin status. Patients were otherwise retained for descriptive reporting when possible.

Prostate cancer Grade Groups were assigned according to the International Society for Urological Pathology grade group system [20]. Risk stratification was performed using the National Comprehensive Cancer Network (NCCN) Guidelines for Prostate Cancer [21]. BCR was defined as a rise in PSA in two consecutive measurements, with the last measurement ≥ 0.2 ng/mL [22].

Erectile function was defined as the ability to achieve an erection firm enough for penetration in at least half the attempts, with or without the use of PDE5i. Time to return of erectile function was calculated as the number of days between the date of surgery and the first clinician note or SHIM score describing patient-reported recovery. SHIM scores were categorized as follows: 22–25 indicated no erectile dysfunction (ED), 17–21 mild ED, 12–16 mild-to-moderate ED, 8–11 moderate ED, and 5–7 severe ED [23].

Urinary continence was defined as being completely pad-free, and time to continence was calculated as the number of days between urethral catheter removal and the first clinician note documenting patient-reported continence. AUA-SS was used to evaluate lower urinary tract symptoms and was interpreted as follows: 0–7 mild, 8–19 moderate, and 20–35 severe symptoms [24]. Postoperative AUA-SS and SHIM scores were typically collected at the three-month follow-up visit.

The primary outcomes of this study were time to recovery of urinary continence and erectile function following SP-TV-RARP. Secondary outcomes included positive surgical margin rate, biochemical recurrence, and perioperative measures such as operative time, estimated blood loss, length of stay, and complication rates.

Basic descriptive statistics were calculated for preoperative, intraoperative, and postoperative variables. Categorical variables were summarized using counts and percentages, while continuous variables were summarized using medians and interquartile ranges due to non-normal distribution and small sample size. Pre- and post-operative SHIM and AUA-SS were compared using the Wilcoxon signed-rank test. Differences in preoperative SHIM scores between patients who did and did not regain erectile function were assessed using the Wilcoxon rank-sum test. Positive surgical margin (PSM) rates were compared between patients with organ-confined disease (≤pT2) and those with extraprostatic extension (≥pT3) on final pathology. Fisher’s exact test was used to assess statistical significance. Both odds ratios (OR) with exact 95% confidence intervals (CIs) and relative risks (RR) with 95% CIs were calculated to quantify the association between pathologic stage and PSM risk.

Time to urinary continence and erectile function recovery was plotted using Kaplan–Meier failure curves to visualize patterns of functional return. Patients who had not regained urinary continence or erectile function at their last documented urology or radiation oncology follow-up visit were censored at that time point. These curves were intended as descriptive visualizations rather than formal survival analyses.

All statistical analyses and figure generation were performed using Stata/BE 18.0 (StataCorp LLC, College Station, TX, USA). This study was approved by the Yale University Institutional Review Board (IRB# 2000040698) with a waiver of informed consent and HIPAA authorization due to its retrospective design and minimal risk. All protected health information was handled in compliance with institutional and federal regulations.

## 3. Results

### 3.1. Baseline Functional Characteristics

A total of 21 patients underwent SP-TV-RARP during the study period, with a median follow-up duration of 3.5 months (IQR 2.3–4.4). The cohort had a median patient age of 65 years and a median BMI of 27.6. The median preoperative PSA level was 6.6 ng/mL (IQR 5.1–8.4), and the median prostate volume on MRI was 40 mL (IQR 32.0–43.9). Most patients had a PIRADS 4 lesion on MRI (75%), Grade Group 2 disease on biopsy (85.7%), and favorable intermediate-risk disease (85.7%). The median baseline AUA-SS was 9 (IQR 6–19), and the median baseline SHIM score was 21 (IQR 11–24). Approximately half of patients reported no erectile dysfunction, and 52.4% of patients were using a PDE5i preoperatively for either pre-existing ED or as part of penile rehabilitation preparation (Table 1).

### 3.2. Perioperative and Postoperative Outcomes

The median operative time was 250 min (IQR 240–300, range 200–360), and the median EBL was 300 mL (IQR 200–500, range 50–900). No intraoperative complications or conversions to multiport or open approaches were reported. A total of 9 patients (42.9%) were discharged home on the day of surgery, and 12 patients (57.1%) were discharged on postoperative day 1. All patients had their urethral catheter removed on post-operative day 7. No post-operative complications were reported within 30 days of the surgery date (Table 2).

### 3.3. Functional Outcomes

A total of 20 patients (95.2%) had a follow-up appointment with a urologist at the time of data collection. Patients were typically seen 7 days postoperatively for urethral catheter removal and scheduled for follow-up visits at approximately 3 and 6 months. Exact follow-up intervals varied by patient, with some seen with varying schedules based on patient factors.

**Figure 1:**
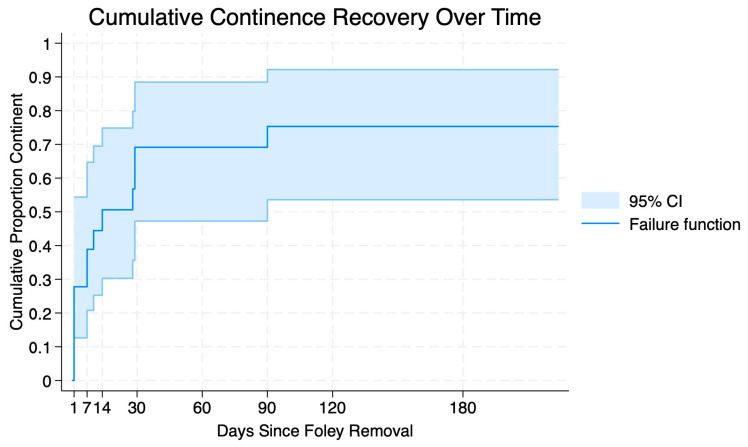
Inverse Kaplan–Meier curve depicting cumulative urinary continence recovery following urethral catheter removal. The shaded areas represent 95% confidence intervals.

A total of 14 patients were fully continent at their most recent appointment (70.0%). Among patients who achieved urinary continence, 6 (42.9%) patients were continent within one day of urethral catheter removal, 10 (71.4%) within two weeks, 13 (93.0%) within one month, and 14 (100%) within 3 months. The median time to continence was 7 days (IQR 1–24.5; range 1–90) (Figure 1).

A total of 5 patients underwent the procedure within 3 months of data analysis and thus were not included in the 3-month post-operative measures. Of the remaining 16 patients, 12 patients (75%) were pad-free following urethral catheter removal, and 4 patients (26.7%) were using one to two pads per day at 3 months (Table 3).

**Figure 2:**
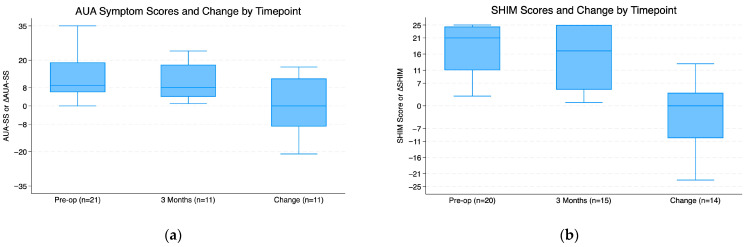
Box-And-Whisker plots showing pre-operative, 3-month, and change in scores. Plots show median, interquartile range, minimum, and maximum. (**a**) AUA symptom scores; (**b**) SHIM scores.

At 3 months postoperatively, the median change in SHIM score from baseline was 0 (IQR −9.75 to 3), and the median change in AUA-SS was 0 (IQR −6.5 to 9). There is no statistically significant difference in SHIM scores (*p* = 0.73) or AUA-SS (*p* = 1.00) compared to baseline (Figure 2).

**Figure 3:**
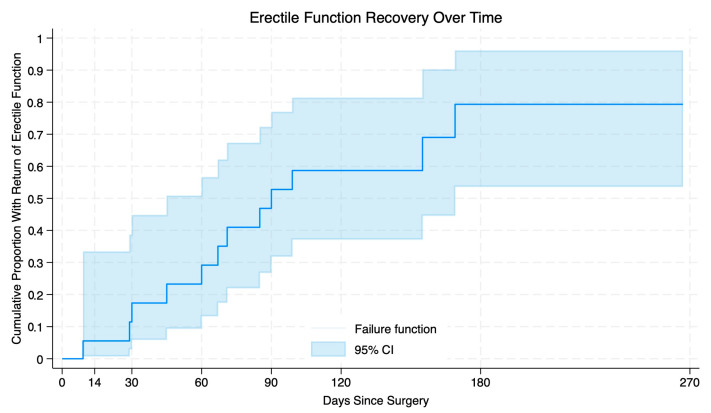
Inverse Kaplan–Meier curve depicting cumulative erectile function recovery following surgery. The shaded area represents 95% confidence intervals.

Erectile function data were available for 18 patients at their most recent follow-up. A total of 12 patients (66.7%) achieved erections firm enough for penetration, with a median time from the date of surgery to erectile function of 69 days (IQR 41.25–92.25, range 29–150) (Table 3). A Kaplan–Meier curve illustrating time to return of erectile function is shown in Figure 3. The median preoperative SHIM score was 21 among those who regained erectile function and 14 among those who did not.

### 3.4. Oncologic Characteristics and Outcomes

The median pre-operative PSA was 6.4 ng/mL with a range of 3.1 to 11.5 ng/mL. Median prostate volume on pre-operative MRI was 40 cc (IQR 32–43.9, range 19–54). Following prostate biopsy, 18 patients (85.7%) had Grade Group 2 disease. The majority of patients had favorable intermediate-risk disease (18 patients, 85.7%).

Pathologic staging revealed organ-confined disease (≤pT2) in 10 patients (47.6%) and extraprostatic extension (≥pT3) in 11 patients (52.4%). Seminal vesicle invasion was present and bilateral in 2 patients (9.5%). Only patients who declined pelvic lymph node dissection were eligible for the transvesical approach.

Positive surgical margins were noted in 13 patients (62%). When stratified by pathologic stage, positive surgical margins were noted in 30% of patients with pT2 disease and 90.9% of those with pT3 disease. Patients with pT3 disease were approximately three times more likely to have a positive margin (RR = 3.03, 95% CI 1.15–7.95, *p = 0.008*).

Median PSA follow-up time, defined as the interval from surgery to the most recent postoperative PSA lab, was 3 months (IQR 0–6 months). Biochemical recurrence occurred in 2 patients (15.4%) (Table 4).

## 4. Discussion

In this initial single-institution experience with SP-TV-RARP, we observed rapid recovery of urinary continence and erectile function, with the majority of patients regaining these functions within three months. The procedure was associated with no intraoperative or early postoperative complications, but a higher rate of positive surgical margins, particularly in pT3 disease, was noted during the early adoption phase.

Robot-assisted radical prostatectomy (RARP) remains one of the most common treatments chosen for localized prostate cancer, making up an estimated 80–90% of all RP cases in the United States, with the rate expected to continue to rise [2,25]. Postoperative urinary continence and erectile function are key patient-reported quality-of-life outcomes for patients following the procedure. Traditional multi-port RARP yields excellent long-term oncologic control as well as recovery of urinary continence and erectile function. Given such outstanding overall results, attention has begun to focus on shortening the recovery time. In this framework, the introduction of the single-port robotic approach has led to the exploration of various surgical techniques with the aim of improving functional outcomes while maintaining similar oncologic outcomes.

Most recently, one surgeon at our institution has adopted the SP-TV-RARP. This novel approach, pioneered by Kaouk et al., has been associated with decreased post-operative opioid requirements, shorter hospital stays, and reduced urethral catheter duration [6]. Most comparative data from North American series have come from the same pioneering group. In a recent propensity-matched analysis, Soputro et al. reported that the SP transvesical approach was associated with higher rates of same-day discharge, reduced opioid prescriptions, shorter catheter duration, and improved early continence recovery, defined as 0 pads per day, compared with the multiport transperitoneal approach [26]. Another matched-pair study from the same group found faster early continence recovery with the transvesical approach compared with the extraperitoneal single-port technique, with no differences in oncologic outcomes [27]. While encouraging, these findings are from retrospective, single-institution experiences, underscoring the importance of independent evaluation. The current study provides the first independent confirmation of results of the aforementioned publications.

In the current analysis of SP-TV-RARP, we found that the approach shows promising early functional recovery of urinary continence and erectile function. Our data support prior findings suggesting that the transvesical approach may offer faster recovery of urinary continence and erectile function compared to traditional transperitoneal multi-port techniques [3,28,29].

Using the strictest definition of being pad-free, urinary continence recovery was notably rapid in our cohort: 43% of patients were continent within 24 h of urethral catheter removal, and 72% achieved continence within two weeks. The plotted time-to-recovery curve revealed a steep early continence recovery, with 30% of all patients continent immediately following catheter removal and a median time to continence of 7 days. These results are comparable to outcomes reported in prior SP-TV-RARP series. For example, Ramos-Carpinteyro et al. observed that 62% of all patients recovered urinary continence at 2 weeks and 77% at 3 months, nearly identical to our findings of 67% and 75%, respectively [17]. Data from traditional multiport RARP series often report urinary continence rates of 69–96% at 12 months [30]. Our findings suggest that early continence recovery may be accelerated through a combination of the Retzius-sparing approach and reduced abdominal and pelvic manipulation inherent to the transvesical single-port approach.

Erectile function recovery was similarly encouraging. Among patients who regained erections sufficient for penetration, the median time to return of erectile function was 69 days, with two-thirds of the entire cohort achieving return of erectile function within 6 months. This is notable given that 30% of patients reported moderate or severe ED preoperatively. In the multiport RARP series, erectile function recovery often occurs over a longer time frame [29,30]. For instance, Arezki et al. found a median time to return of erectile function of 853 days for patients in a similar age range to this study’s patients, representing outcomes currently observed with established techniques [28].

Regarding oncologic outcomes, our study observed a 62% overall positive surgical margin (PSM) rate. Although this is concerning, this elevated rate likely reflects the high proportion of pT3 disease in our cohort (52.4%), which is associated with greater tumor burden and extraprostatic extension, and technical considerations unique to the transvesical approach. Even for surgeons experienced with single-port RARP, the transvesical route involves different dissection planes, a more confined working space, and altered visualization compared to extraperitoneal or transperitoneal approaches. These factors could add complexity during early adoption, as early adoption has been associated with higher PSM rates [31]. However, the markedly higher PSM rate in pT3 versus pT2 disease (90.9% vs. 30%), with over a threefold higher risk, aligns with multiport literature and suggests that extent of disease may be a more significant contributor [32]. These findings underscore the importance of thorough preoperative counseling and closer postoperative surveillance for patients with pT3 disease. Unfortunately, in our cohort, preoperative clinical staging and risk stratification did not reliably identify pT3 disease. While the pT stage is only available postoperatively, we encourage surgeons during the learning curve of the transvesical approach to preferentially select patients with favorable preoperative risk factors when possible.

The reproducibility of SP-TV-RARP in anatomically complex settings remains to be fully explored. Our early series excluded patients with larger prostates or the need for lymph node dissection to ensure procedural feasibility during early adoption. Prior reports from the pioneering center have demonstrated that SP-TV-RARP can be successfully performed in patients with challenging anatomy, such as those with extensive prior abdominal surgeries, with favorable perioperative outcomes [33]. However, these series also note potential limitations in such settings, including restricted lymph node dissection, anastomotic tension in previously operated bladders, and increased difficulty with very large prostates or prior pelvic radiation. These factors may influence both functional and oncologic outcomes and should be considered as experience with the approach expands.

Recent European studies highlighting high-volume center experiences with Retzius-sparing multiport RARP have underscored the challenge of balancing early functional recovery with oncologic control [34,35]. Analyses from these cohorts have identified important factors most critical to early oncologic outcomes, including the learning curve with new approaches or technology, patient selection, and nerve-sparing intent, which may all influence positive surgical margin rates. While these findings are in multiport approaches, they provide important context and can translate to single-port platforms where differences in instrument configuration, working space, and tissue handling could provide their own learning curve. To contextualize our findings, a summary of the two previously published SP-TV-RARP series is provided in Supplementary Table S1 [15,36].

This study has several important limitations. First, the small sample size and retrospective, observational, single-institution nature limit the power and generalizability of our findings. All cases were performed by a single surgeon, which, while ensuring procedural consistency, may introduce operator-dependent bias. Additionally, the surgical technique evolved during the study period as experience with SP-TV-RARP increased, including refinements to operative steps and patient selection criteria. Because these modifications were not fully known in advance, a prospective protocol could not be established, and relevant data elements were reviewed retrospectively after the fact. This approach is subject to the inherent limitations of retrospective reviews, including reliance on existing documentation, potential variability in data capture, and inability to control for all confounding variables. Excluding patients with prostate volumes > 60 mL, while consistent with current technical feasibility for SP-TV-RARP, may have introduced a favorable selection bias by limiting the cohort to smaller prostates and potentially more favorable anatomy. Furthermore, the absence of a control group as well as patient selection precludes direct comparison to other approaches, and comparisons to findings in the literature are inherently limited by differences in patient populations and methodology.

Functional and oncologic outcomes were evaluated in the short term, with these cases all occurring within 1 year of data extraction and analysis. This may underestimate oncologic outcomes and exclude long-term complications. Additionally, not all patients had complete data for validated measures such as SHIM, AUA-SS, or PSA, which may affect the accuracy of our reported data. In particular, erectile function outcomes occasionally relied on clinical documentation of subjective return of erections, which limits granularity and interpretation. Finally, the time-to-recovery curves for continence and erectile function were based on the available follow-up and were intended as descriptive visualizations; estimates for patients with shorter follow-up, particularly less than 3 months, should be interpreted cautiously.

In summary, this study represents the first series of SP-TV-RARP from outside the pilot institution, reporting early functional and oncologic outcomes in the setting of early adoption. Our findings demonstrate early return of urinary continence and erectile function with this approach, while also addressing concerns regarding oncologic control during the introduction of novel techniques. These results support the feasibility and safety of SP-TV-RARP and highlight the need for continued evaluation to assess long-term oncologic durability, sustained functional recovery, and broader applicability across diverse practice settings.

## 5. Conclusions

Transvesical single-port robot-assisted radical prostatectomy (SP-TV-RARP) is a novel surgical approach that integrates Retzius-sparing principles with a minimally invasive extraperitoneal technique. In this initial single-institution experience, early functional outcomes appeared favorable, with a majority of patients regaining urinary continence and erectile function within three months of surgery. However, the small sample size, short follow-up, lack of a control group, and high positive surgical margin rate, which may be influenced by both the learning curve and margin location, limit the strength of these findings and preclude definitive comparison to standard multiport approaches. Further prospective studies with larger cohorts, longer follow-up, and comparative designs are warranted to assess the functional and oncologic durability and broader clinical utility of SP-TV-RARP.

## Figures and Tables

**Table 1:** Baseline characteristics with median values and interquartile range or percentage of patients.

Baseline Variable	*n* = 21	Median (IQR)
Age (years)		65 (60–68)
BMI (kg/m^2^)		27.6 (24.4–31.3)
Pre-op Hgb (g/dL)		14.7 (14.2–15.4)
Pre-op Cr (mg/dL)		1 (0.87–1.16)
Baseline AUA-SS		9 (6–19)
Baseline SHIM		21 (11–24.25) (*n* = 20)
Median Pre-operative PSA (range)		6.4 ng/mL (3.1–11.5)
Pre-Operative MRI	*n* = 20	
	1 lesion	16 patients (80%)
	2 lesions	4 patients (20%)
	PIRADS-2	1 patient (5%)
	PIRADS-4	15 patients (75%)
	PIRADS-5	4 patients (20%)
Median Prostate Volume (IQR)		40 mL (32–43.9)
ISUP Grade Group		
	GG1	1 patient (4.8%)
	GG2	18 patients (85.7%)
	GG3	1 patient (4.8%)
	GG4	1 patient (4.8%)
NCCN Risk Stratification		
	Very Low	0 patients (0%)
	Low	1 patient (4.8%)
	Favorable Intermediate	18 patients (85.7%)
	Unfavorable Intermediate	1 patient (4.8%)
	High	1 patient (4.8%)
	Very High	0 patients (0%)

Hgb = Hemoglobin; Cr = Creatinine; AUA-SS = American Urological Association Symptom Score; SHIM = Sexual Health Inventory for Men.

**Table 2:** Perioperative Outcomes and associated median values and standard deviation.

Variable	*n* = 21
Median Operative Time (minutes) (IQR)	250 (240–300)
Median EBL (mL) (IQR)	300 (200–500)
Discharged Same Day	9 patients (42.9%)
Discharged Next Day	12 patients (57.1%)
Intraoperative Complications	0
Postoperative Complications	0

EBL = estimated blood loss.

**Table 3:** Postoperative Functional Outcomes.

Variable		Number of Patients (%)
Baseline SHIM		20 patients
	No ED	10 (50%)
	Mild	3 (15%)
	Mild-to-Moderate	1 (5%)
	Moderate	4 (20%)
	Severe	2 (10%)
3-Month SHIM		15 patients
	No ED	5 (33.3%)
	Mild	3 (20%)
	Mild-to-Moderate	3 (20%)
	Moderate	0 (0%)
	Severe	4 (26.7%)
Median Change SHIM (IQR)	0 (−9.75, 3)	
Return of Erectile Function at Last Visit	12 (66.7%)	
Median Time to Return of Erection (IQR)	69 days (41.25, 92.25)	
Baseline AUA-SS		21 patients
	No Symptoms	1 (4.8%)
	Mild	7 (33.3%)
	Moderate	8 (38.1%)
	Severe	5 (23.8%)
3-Month AUA-SS		11 patients
	No Symptoms	0 (0%)
	Mild	5 (45.5%)
	Moderate	5 (45.5%)
	Severe	1 (9.1%)
Median Change AUA-SS (IQR)	0 (−6.5, 9)	
Continent at Last Visit	14 (70.0%) (*n* = 20)	
Continence Timing	*n* = 20	
	Within 1 day	6 (30%)
	Within 7 days	8 (40%)
	Within 14 days	10 (50%)
	Within 30 days	13 (65%)
	Within 90 days	14 (70%)
Number of Pads at 3 months	n = 16	
	0 pads/day	12 (75%)
	1–2 pads/day	4 (25%)
Median Time to Continence (IQR)	7 days (1, 24.5)	

**Table 4:** Oncologic and Pathologic Outcomes.

Variable		*n* = 21
Median Prostate Weight (IQR)		40 g (34–44)
Pathologic Staging		
	pT2	6 patients (28.6%)
	pT2c	4 patients (19.0%)
	pT3a	9 patients (42.9%)
	pT3b	2 patients (9.5%)
Seminal Vesicle Invasion	2 patients (9.5%)	
Perineural Invasion		20 patients (95%)
	Extensive	7 patients (35%)
Positive Surgical Margins		13 patients (62%)
	Of those with pT2	3 patients (30%)
	Of those with pT3	10 patients (90.9%)
Median Follow-Up PSA (IQR)		3 months (0–6)
	3 months	0 (0–0.05)
	6 months	0 (0–0.08)
Biochemical Recurrence	2 patients (15.4%)	4 (25%)
Median Time to Continence (IQR)	7 days (1, 24.5)	

## Data Availability

The data presented in this study are available on request from the corresponding author due to ongoing data collection and a small patient population.

## Supplementary Materials

The following supporting information can be downloaded at: https://www.mdpi.com/article/10.3390/cancers17172793/s1, Table S1: Summary of published SP-TV-RARP series.

## Abbreviations

The following abbreviations are used in this manuscript:

RP	Radical Prostatectomy
RARP	Robot-Assisted Radical Prostatectomy
SP-RARP	Single-Port Robot-Assisted Radical Prostatectomy
LPV	Laparoscopy with Pneumovesicum
SHIM	Sexual Health Inventory for Men
AUA-SS	American Urological Association Symptom Scores
NCCN	National Comprehensive Cancer Network
BCR	Biochemical Recurrence
PSA	Prostate-Specific Antigen
PSM	Positive Surgical Margins
PDE5i	Phosphodiesterase Type 5 Inhibitor
ED	Erectile Dysfunction
BMI	Body Mass Index
IQR	Interquartile Range
EBL	Estimated Blood Loss
