# Supplementary material for: Early Continence and Erectile Function Recovery Following Transvesical Single-Port Robot-Assisted Radical Prostatectomy: Initial Single Institution Experience
Source: Cancers (Basel). 2025 Aug 27;17(17):2793. doi: 10.3390/cancers17172793 (PMC12427196; doi:10.3390/cancers17172793)
Supplement: Supplementary file 1 [file cancers-17-02793-s001.zip › cancers-3820358-supplementary.pdf]

# Supplementary Materials: Early Continence and Erectile Function Recovery Following Transvesical Single-Port Robot-Assisted Radical Prostatectomy: Initial Single Institution Experience

**Brandon L. Ward [1,2], Anthony Y. Zhang [1,3], Michael S. Leapman [1,4], Jaime A. Cavallo [1,4] and Isaac Y. Kim [1,*]**

**Table S1.** Summary of published SP-TV-RARP series.

| Study | Sample Size | Continence Definition | Return to Continence | Return of Erectile Function | Percent Extraprostatic Extension | Positive Surgical Margin Rate | Follow Up |
|---|---|---|---|---|---|---|---|
| Ramos-Carpinteyro et al., 2023[1] | 100 | 0-1 pad | 50% within 3 days | 37% mild-to-moderate ED after 6 months | 38% | 15.0% | Median 10.4 months |
| Zhou et al., 2020[2] | 35 | 0-1 pad | 91.4% immediately | Mean IIEF-5 18 (mild ED) at 12 months | 0% | 11.4% | ≥ 12 months |
| Present Study | 21 | 0 pads | 43% immediately | 66.7% moderate or better ED at 3 months | 52.4% | 62.0% | Median 3.5 months |

Of note, Ramos-Carpinteyro et al 2023 [1] included patients described in Kaouk et al 2021[3]. Immediate continence notes urinary continence following urethral catheter removal postoperatively.

**Supplemental References**

1. Ramos-Carpinteyro, R.; Ferguson, E.L.; Chavali, J.S.; Geskin, A.; Kaouk, J. First 100 Cases of Transvesical Single-Port Robotic Radical Prostatectomy. *Asian J. Urol.* **2023**, *10*, 416–422, doi:10.1016/j.ajur.2022.12.005.
2. Zhou, X.; Fu, B.; Zhang, C.; Liu, W.; Guo, J.; Chen, L.; Lei, E.; Zhang, X.; Wang, G. Transvesical Robot-assisted Radical Prostatectomy: Initial Experience and Surgical Outcomes. *BJU Int.* **2020**, *126*, 300–308, doi:10.1111/bju.15111.
3. Kaouk, J.; Beksac, A.T.; Abou Zeinab, M.; Duncan, A.; Schwen, Z.R.; Eltemamy, M. Single Port Transvesical Robotic Radical Prostatectomy: Initial Clinical Experience and Description of Technique. *Urology* **2021**, *155*, 130–137, doi:10.1016/j.urology.2021.05.022.
